# Machine learning‐based prediction of atrial fibrillation in patients with atrial high‐rate episodes

**DOI:** 10.1111/eci.70121

**Published:** 2025-09-14

**Authors:** Amir Askarinejad, Tommaso Bucci, Niloofar Asgharzadeh, Zahra Amirjam, Enrico Tartaglia, Michele Rossi, Yang Chen, Yalin Zheng, Gregory Y. H. Lip, Majid Haghjoo

**Affiliations:** ^1^ Liverpool Centre for Cardiovascular Sciences at University of Liverpool, Liverpool John Moores University and Liverpool Heart & Chest Hospital Liverpool UK; ^2^ Department of Clinical Internal, Anesthesiologic and Cardiovascular Sciences Sapienza University of Rome Rome Italy; ^3^ Cardiac Electrophysiology Research Center Rajaie Cardiovascular Institute Tehran Iran; ^4^ Department of Cardiology Ardabil University of Medical Sciences Ardabil Iran; ^5^ Department of Clinical Medicine Aalborg University Aalborg Denmark; ^6^ Medical University of Bialystok Bialystok Poland; ^7^ Department of Electrophysiology Rajaie Cardiovascular Institute Tehran Iran

**Keywords:** asymptomatic atrial fibrillation, atrial fibrillation, atrial high rate episodes, machine learning, sub‐clinical atrial fibrillation

## Abstract

**Background:**

Given the modest performance of available predictive models in estimating the risk of atrial fibrillation (AF) in patients with atrial high‐rate episodes (AHREs) detected by cardiac implantable electronic devices (CIEDs), this study explores the potential use of machine learning (ML) algorithms in this context.

**Purpose:**

To assess the ability of ML techniques in identifying patients with AHRE at high risk of AF.

**Methods:**

In this prospective study, we enrolled patients without a prior history of AF who experienced at least one AHRE episode detected by CIEDs. ML techniques were applied to predict the 1‐year risk of developing new‐onset AF based on the following variables: age, BMI, sex, smoking, hypertension, diabetes, coronary artery disease, chronic kidney disease, dyslipidaema, history of stroke or transient ischaemic attack, vascular heart disease, left atrial enlargement (LAE) and congestive heart failure.

**Results:**

Study population consists of 100 patients (48% male, mean age 66.0 ± 18.0 years), of whom 24 developed AF (24%) after 1‐year follow‐up. The CatBoost ML model achieved the highest AUC (.857, 95% CI .671–.999) when compared to other ML models and all clinical risk scores. The top four most influential predictors of AF in the CatBoost model were LAE, hypertension, diabetes and age.

**Conclusions:**

ML techniques are robust in predicting AF in patients with AHREs. Further validation in larger, independent cohorts is warranted.

## Graphical abstract

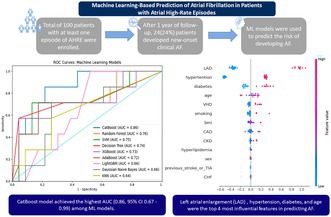

## 1 INTRODUCTION

Atrial high rate episodes (AHREs) are defined as periods of rapid atrial activity with an average heart rate exceeding 175 beats per minute, lasting from at least 5 min to a maximum of 23 h and 59 min, with a reported prevalence ranging from 30% to 70%.^1^, ^2^ Noteworthy, AHREs have been associated with an increased risk of stroke, systemic embolism and mortality.^3^ This increased risk appears to stem from a higher likelihood of progression to atrial fibrillation (AF).^4^, ^5^

Given this association, it has been hypothesized that patients with AHRE might benefit from indiscriminate treatment with oral anticoagulants (OACs). However, randomized clinical trials investigating the efficacy of OACs in this population yielded conflicting results.^6^, ^7^ These findings highlight the need for more robust risk stratification tools to identify high‐risk AHRE patients who may benefit from closer monitoring or preventive antithrombotic strategies.

In recent years, several clinical risk scores including CHA_2_DS_2_‐VASc, C_2_HEST, mCHEST, HAT_2_CH_2_ and HAVOC scores have been employed to assess the risk of new‐onset AF in the general population. However, their predictive performance in AHRE patients has been modest, limiting their utility in clinical practice.^8^ Conversely, while machine learning (ML) prediction models have demonstrated superior predictive capabilities in various clinical settings for identifying patients at high risk of developing AF,^9^, ^10^ data on their application in patients with AHREs remain limited.

Therefore, in this study we aimed to: (i) assess the predictive role of ML models in identifying AHRE patients at high risk of incident AF, and (ii) compare the predictive value of ML models with traditional risk scores in an exploratory analysis.

## 2 METHODS

### 2.1 Study design and settings

This was a prospective cohort study conducted at the Electrophysiology Department of Rajaie Cardiovascular Medical and Research Center. Consecutive patients with CIEDs referred between November 2019 and July 2021 were considered for enrollment. Eligible participants were aged ≥18 years and had experienced at least one AHRE, defined as a period of atrial activity with an average rate exceeding 175 beats per minute and lasting from a minimum of 5 min to a maximum of 23 h and 59 min [1]. Patients with a prior diagnosis of clinical AF or with AF‐related symptoms such as palpitations, dizziness, syncope or shortness of breath, were excluded. The study protocol was approved by the Ethics Committee of the Rajaie Cardiovascular Medical and Research Center (Ethics approval ID: IR.IUMS.REC.21315), and written informed consent was obtained from all participants in accordance with the Declaration of Helsinki.

### 2.2 Baseline data collection

At the time of enrollment, demographic and clinical data were obtained from medical records. These included age, sex, smoking status, comorbid conditions including hypertension, diabetes, coronary artery disease (CAD), chronic kidney disease (CKD), dyslipidaemia, history of stroke or transient ischaemic attack (TIA), vascular heart disease and type of CIED, along with the indication for device implantation. Echocardiographic parameters were also recorded, including left atrial size and left ventricular ejection fraction (LVEF).

### 2.3 Echocardiographic assessment

Transthoracic echocardiography was performed by experienced fellows using a Philips Epiq 7c ultrasound system. LVEF was calculated using the modified Simpson's biplane method in accordance with recommendations from the American Society of Echocardiography and the European Association of Cardiovascular Imaging.^11^, ^12^

Left atrial size was quantified as the left atrial volume index, calculated by dividing left atrial volume by body surface area. Left atrial enlargement (LAE) was classified according to guideline‐defined thresholds: normal (≤34 mL/m^2^), mild LAE (35–41 mL/m^2^), moderate LAE (42–48 mL/m^2^) and severe LAE (≥48 mL/m^2^)^11^ and was used as a categorical variable in the ML prediction models.

### 2.4 Clinical risk scores

Several established clinical risk scores were calculated at baseline to allow an exploratory comparison with ML models. These included the C_2_HEST, HAVOC, CHADS_2_, CHA_2_DS_2_–VASc and HATCH scores, using their original definitions and point allocations as described in the literature.^13^, ^14^, ^15^, ^16^

### 2.5 Study outcomes

The primary outcome was the development of clinical AF during the one‐year follow‐up period, confirmed by either a 12‐lead electrocardiogram (ECG) or a single‐lead ECG tracing lasting at least 30 s. AF was diagnosed based on the presence of irregular R‐R intervals, the absence of distinct repeating P waves and irregular atrial activity.^17^ The primary aim was to evaluate the predictive performance of ML models in predicting the occurrence of clinical new‐onset AF in patients with AHRE. The secondary aim was to compare the performance of ML‐based approaches with that of conventional clinical risk scores in this population.

### 2.6 Statistical analysis

#### 2.6.1 Descriptive analysis

Descriptive statistics were used to summarise the baseline characteristics. Categorical variables were reported as frequencies and percentages, while continuous variables were expressed as means with standard deviations or medians with interquartile ranges, as appropriate. The chi‐square test was applied to assess differences between categorical variables and the independent *t*‐test and Mann–Whitney *U* test were used for comparing normally and non‐normally distributed continuous variables, respectively.

### 2.7 Data Preparation and Preprocessing

#### 2.7.1 Data Preprocessing

No outlier removal, normalisation or feature scaling was performed; categorical variables were handled internally by the algorithms, and no missing data were observed in the dataset.

#### 2.7.2 Collinearity assessment

**FIGURE 1:**
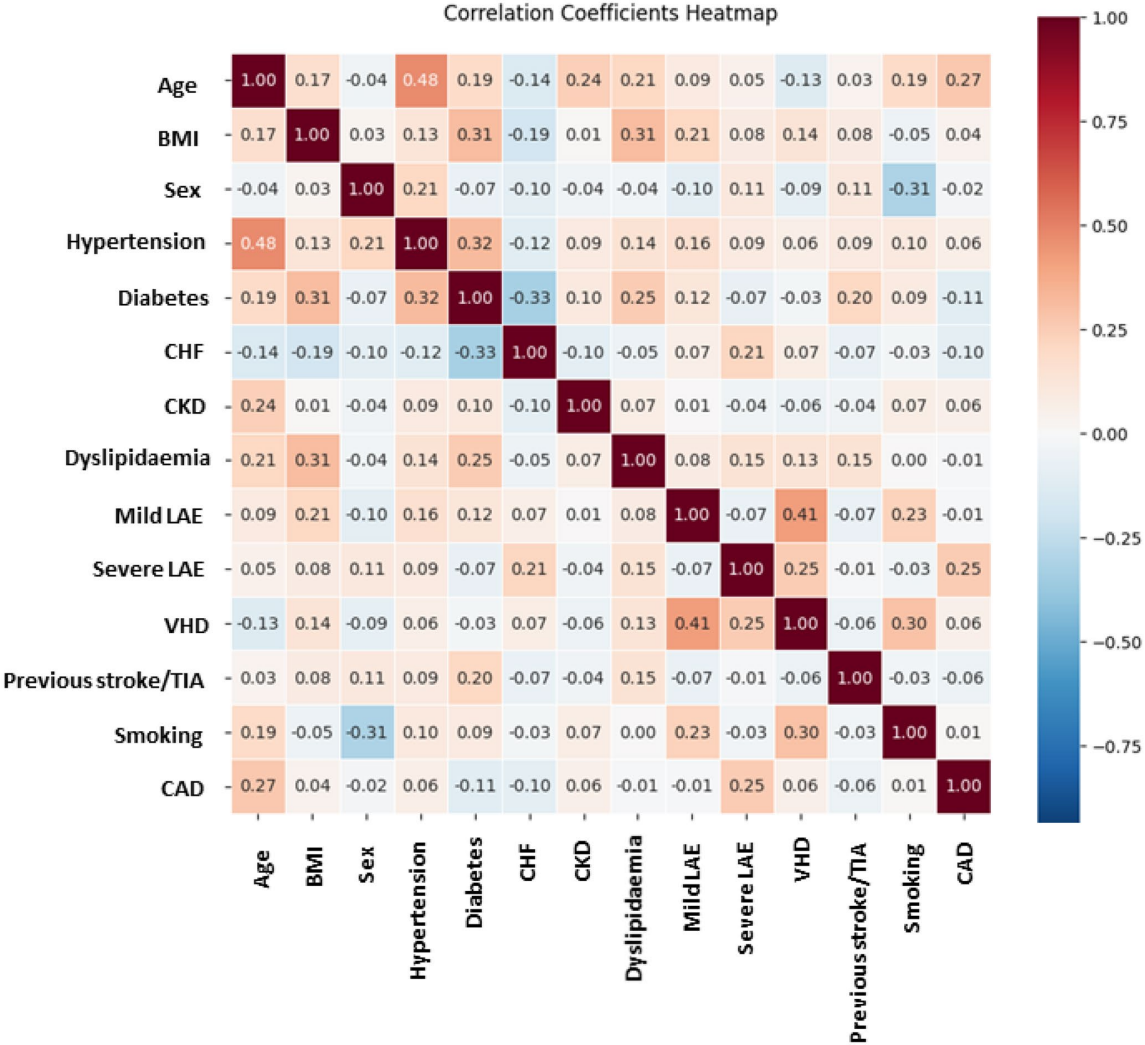
Pearson correlation heatmap of predictors. BMI, Body mass index; CAD, Coronary artery disease; CHF, Congestive heart failure; CKD, Chronic kidney disease; LAE, Left atrial enlargement; TIA, Transient ischemic attack; VHD, Vascular heart disease.

Before conducting the predictive analysis, Pearson correlation coefficients were calculated for all feature pairs and demonstrated in a heatmap (Figure 1). No pair of predictors exceeded an absolute correlation of .8, suggesting that multicollinearity was unlikely to compromise model stability (Figure 1).

#### 2.7.3 Feature selection

Features were chosen based on established AF risk factors from recent clinical guidelines,^17^ as well as on predictors of AHRE progression to AF identified in the literature.^18^ Features that were included in the ML models were age, sex, BMI, smoking, hypertension, diabetes, coronary artery disease, CKD, dyslipidaemia, history of stroke or TIA, vascular heart disease, LAE and congestive heart failure.

### 2.8 Clinical risk‐scores performance

After randomly splitting the data using a 7:3 ratio stratified by AF outcome (random seed = 42), for all clinical risk scores including CHA_2_DS_2_‐VASc, C_2_HEST, HAVOC, CHADS_2_ and HATCH, the optimal cut‐off was calculated according to the maximum Youden index (sensitivity + specificity – 1) analysing the training set (Table S1). Subsequently, accuracy, sensitivity, specificity, positive predictive value (PPV), negative predictive value (NPV), F1‐score and AUC of each clinical risk score in predicting AF were reported, analysing the test set.

### 2.9 Machine‐learning model performance

The dataset was randomly split into training and test sets using a 7:3 ratio to ensure robust performance evaluation. A range of supervised ML algorithms was implemented to predict incident clinical AF, including CatBoost, Random Forest, LightGBM, Support Vector Machine (SVM), XGBoost, k‐Nearest Neighbours (KNN), Decision Tree, AdaBoost and Gaussian Naïve Bayes. Hyperparameters for each model were tuned via stratified 5‐fold cross‐validation, optimising the F1 score. The probability threshold was fixed at .50 for all ML models. Model performance was evaluated using the following metrics: accuracy, sensitivity, specificity, PPV, NPV, F1‐score and AUC. Performance metrics were calculated for each model on the test set. Feature importance analysis was conducted for the best‐performing model to identify the most influential predictors using SHAP (SHapley Additive exPlanations). Receiver operating characteristic curve analysis was performed for all ML models and clinical risk scores, and their AUCs were compared using the DeLong test.^19^

### 2.10 Software, packages and libraries

Descriptive analysis was performed using IBM SPSS statistics software version 29.0.2.0. All prediction analyses were performed using Python version 3.10. Key Python libraries included *scikit‐learn classifiers* (RandomForestClassifier, AdaBoostClassifier, DecisionTreeClassifier, SVC, KNeighborsClassifier and GaussianNB), *XGBoost* (XGBClassifier), *LightGBM* (LGBMClassifier) and *CatBoost* (CatBoostClassifier) for model development; *Optuna* for hyperparameter tuning; *scikit‐learn's permutation_importance* and *SHAP* for feature‐importance analysis; and *matplotlib* for visualization.

## 3 RESULTS

### 3.1 Baseline characteristics of the overall population

Among the 136 patients with AHRE initially evaluated for this study, 100 patients (mean age 66.0 ± 18.00 years; 48% male) were included in the final analysis (Figure S1). Overall, 63% had hypertension, 28% had diabetes mellitus, 19% had CAD, and 8% had heart failure. The mean CHA_2_DS_2_‐VASc score in the study population was 2.3 ± .9. Baseline characteristics, comorbidities, echocardiographic findings and clinical risk scores, including HAVOC, CHADS_2_, HATCH, C_2_HEST and CHA_2_DS_2_‐VASc, are summarised in Table 1.

**TABLE 1:** Comparison of demographic characteristics, comorbidities, device implantation indications, CHA_2_DS_2_‐VASc score and echocardiographic findings between patients who did and did not progress to atrial fibrillation.

Variable		Total study population (*N* = 100)	Patients progressed to AF (*N* = 24)	Patients without progress to AF (*N* = 76)	*p*‐Value
Demographics					
Age		66.0 ± 18.0	71.4 ± 12.7	64.3 ± 19.1	.015
Male		48 (48.0)	12 (50.0)	36 (47.4)	.822
BMI		28.1 ± 5.9	29.2 ± 3.3	28.4 ± 5.4	.409
Comorbidities and echocardiographic findings					
Hypertension		63 (63.0)	22 (91.7)	41 (53.9)	.001
Diabetes mellitus		28 (28.0)	10 (41.7)	18 (23.7)	.003
Dyslipidaema		35 (35.0)	11 (45.8)	24 (31.6)	.041
Smoking		8 (8.0)	4 (16.7)	4 (5.3)	.030
Heart failure		8 (8.0)	4 (16.7)	4 (5.3)	.030
CKD		14 (14.0)	4 (16.7)	10 (13.2)	.336
CAD		19 (19.0)	7 (29.2)	12 (15.7)	.010
Vascular heart disease		18 (18.0)	8 (33.3)	10 (13.2)	.006
LVEF		45.3 ± 9.0	44.2 ± 9.2	45.6 ± 9.0	.580
Device type					
PPM		87 (87.0)	18 (75.0)	69 (90.8)	.109
ICD		4 (4.0)	2 (8.3)	2 (2.6)	
CRT		9 (9.0)	4 (16.7)	5 (6.6)	
Indications of device implantation					
SSS		15 (15.0)	4 (16.7)	11 (14.5)	.333
AVB		81 (81.0)	18 (75.0)	63 (82.9)	
Primary prevention		1 (1.0)	1 (4.2)	0 (0)	
Secondary Prevention		3 (3.0)	1 (4.2)	2 (2.6)	
Clinical risk scores					
CHA_2_DS_2_‐VASc score	Mean value	2.3 ± .9	2.9 ± .5	2.2 ± 1.0	<.001
	Low risk (0)	5 (5.0)	0 (.0)	5 (6.6)	.005
	Intermediate risk (1)	17 (17.0)	0 (.0)	17 (22.4)	
	High risk (≥2)	68 (68.0)	24 (100.0)	44 (71.0)	
CHA_2_DS_2_ score	Mean value	1.5 ± 1.0	2.2 ± .8	1.3 ± 1.0	<.001
	Low risk (0)	0	0	0	<.001
	Intermediate risk (1)	40 (40.0)	4 (16.7)	36 (47.4)	
	High risk (≥2)	60 (60.0)	20 (83.3)	40 (52.6)	
HAVOC score	Mean value	3.8 ± 2.4	3.3 ± 2.3	5.2 ± 2.4	.001
	Low risk (0–4)	68 (68.0)	10 (41.7)	58 (76.3)	.007
	High risk (≥5)	32 (32.0)	14 (58.3)	18 (23.7)	
C_2_HEST score	Mean value	2.0 ± 1.4	2.7 ± 1.3	1.9 ± 1.4	.011
	Low risk (0–1)	36 (36.0)	6 (25.0)	30 (39.5)	.012
	Intermediate risk (2–3)	49 (49.0)	11 (45.8)	38 (50.0)	
	High risk (≥4)	15 (15.0)	7 (29.2)	8 (10.5)	
HATCH score	Mean value	1.6 ± 1.1	2.1 ± 1.1	1.4 ± 1.1	.010
	Low risk (0–1)	54 (54.0)	10 (41.7)	44 (57.9)	.003
	High risk (≥2)	46 (46.0)	14 (58.3)	32 (42.1)	
Echocardiographic findings					
Normal LAE		63 (63.0)	7 (29.1)	56 (73.7)	.001
Mild LAE		29 (29.0)	15 (62.5)	14 (18.4)	
Moderate LAE		6 (6.0)	1 (4.2)	5 (6.6)	
Severe LAE		2 (2.0)	1 (4.2)	1 (1.3)	
LVEF		45.3 ± 9.0	44.3 ± 9.2	45.6 ± 9.0	.580
First detected AHRE episode duration (minute)		6.00 (6.00–18.00)	870.00 (600.00–1425.00)	6.00 (3.00–7.75)	<.001

Abbreviations: AF, atrial fibrillation; AVB, atrioventricular block; BMI, body mass index; CAD, coronary artery disease; CKD, chronic kidney disease; CRT, cardiac resynchronization therapy; ICD, implantable cardioverter defibrillator; LAE, left atrial enlargement; LVEF, left ventricular ejection fraction; PPM, permanent pacemaker; SSS, sick sinus syndrome.

### 3.2 Clinical characteristics of patients developing AF

After 1 year of follow‐up, 24 patients (mean age 71.4 ± 12.7 years; 50% male) developed clinical new‐onset AF. Advanced age, hypertension, diabetes, dyslipidaemia, heart failure and CAD were significantly more prevalent in patients who developed AF compared to those who did not (Table 1). The mean CHA_2_DS_2_‐VASc score was 2.9 ± .5 in patients who developed AF and 2.2 ± 1.0 in those who did not. AHRE episodes were significantly longer in patients who developed AF (Table 1).

### 3.3 Evaluation of the machine learning models

**FIGURE 2:**
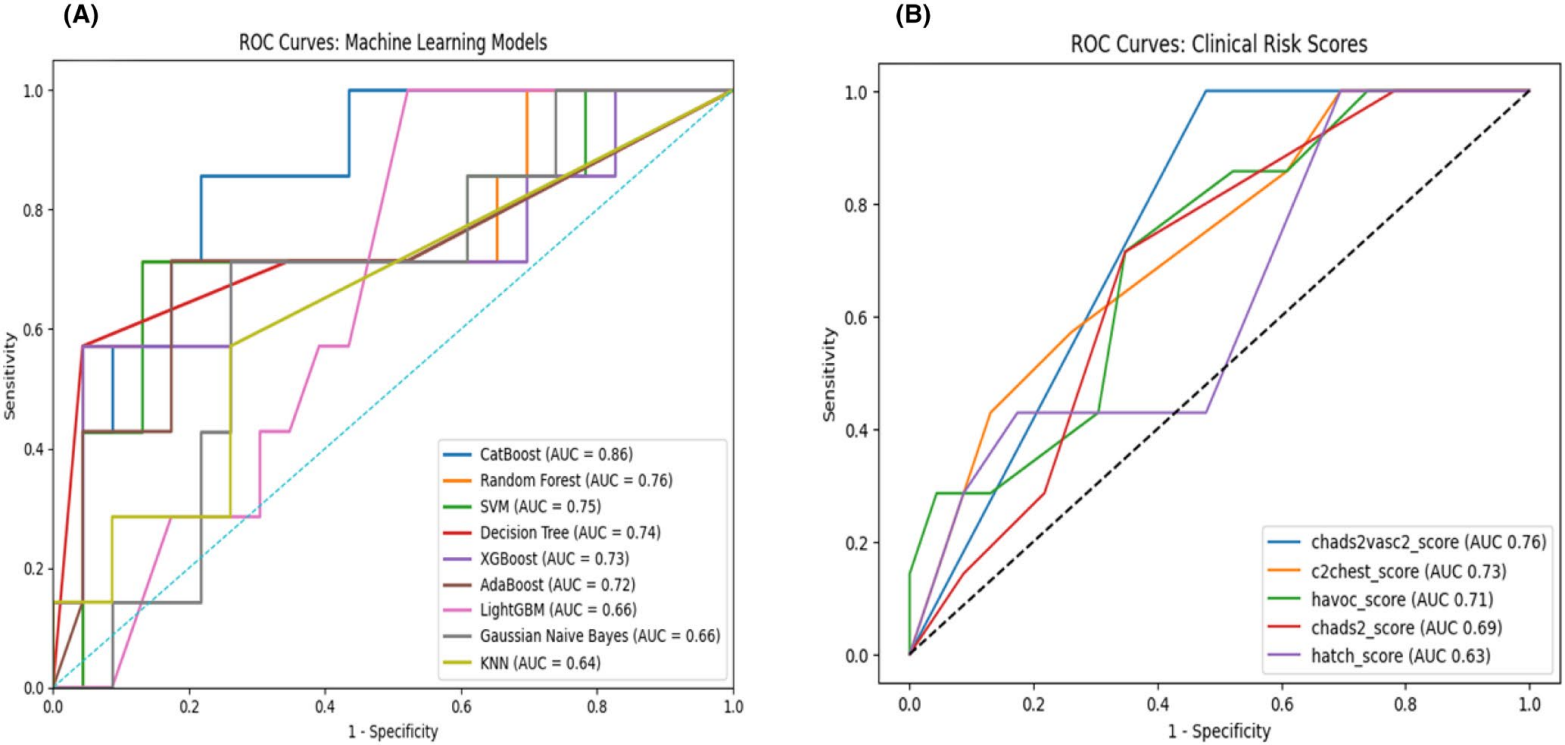
ROC curves of machine learning models (A) and clinical risk scores (B).

**FIGURE 3:**
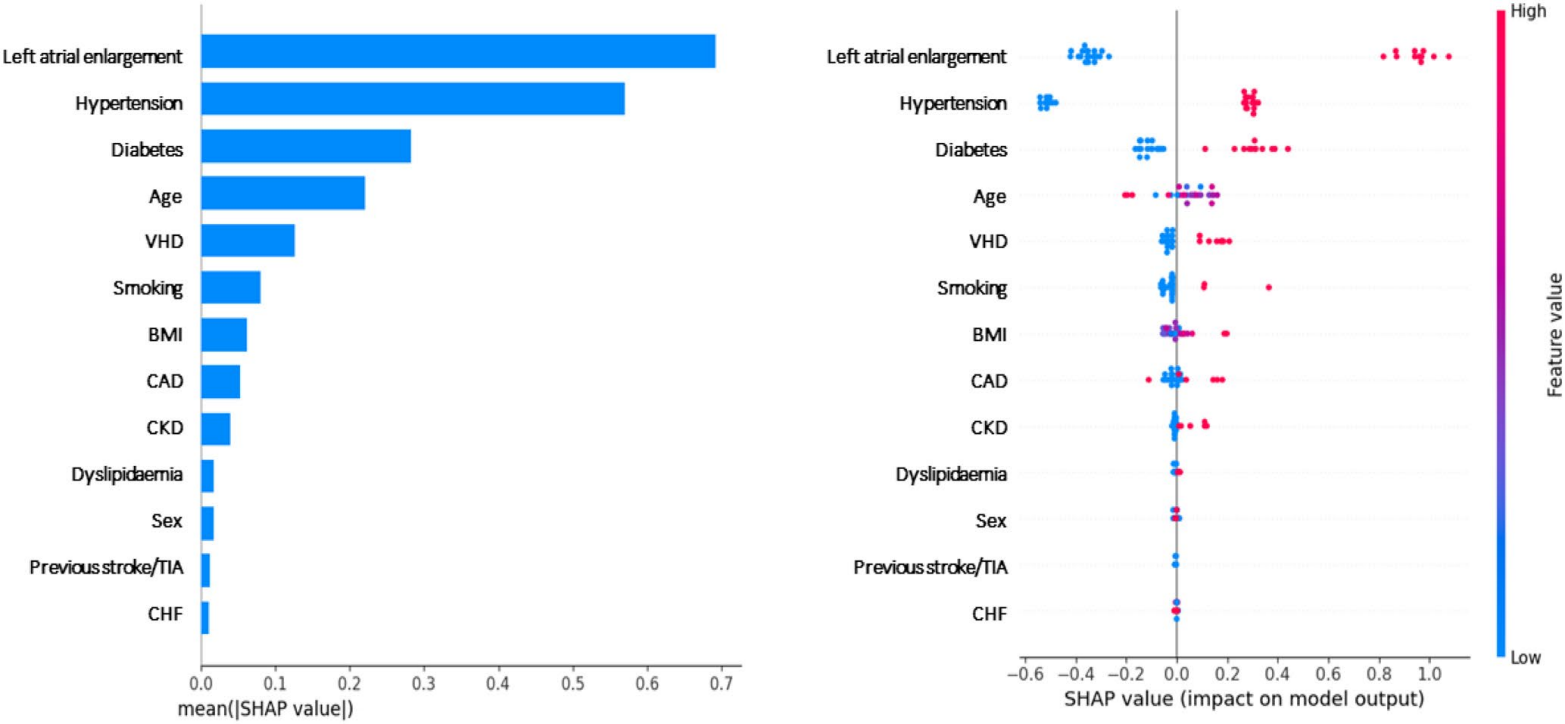
Feature Importance Ranking in the CatBoost Model for Atrial Fibrillation Prediction. BMI, body mass index; CAD, Coronary Artery Disease; CHF, Congestive Heart Failure; CKD, Chronic Kidney disease; LAE, Left Atrial Enlargement; VHD, vascular heart disease.

When analysing the 1‐year risk of AF with different ML approaches, the CatBoost model achieved an accuracy of .800 (95% CI .627–.905), F1‐score of .500 (95% CI .000–.800) and an AUC of .857 (95% CI .671–.999), demonstrating strong overall performance (Figure 2A). Among all models, CatBoost, AdaBoost and SVM achieved the highest accuracy of .800. Table 2 provides a detailed summary of the evaluation metrics for other ML models used in predicting AF. The top four most influential predictors of AF in the CatBoost model were LAE, hypertension, diabetes and age (Figure 3).

**TABLE 2:** Evaluation metrics of machine learning models and clinical risk scores in predicting new‐onset atrial fibrillation.

Model		Accuracy	Sensitivity	Specificity	PPV	NPV	F1‐score	AUC (95% CI)
Machine learning models	CatBoost	.800 (.627–.905)	.429	.913	.600	.840	.500 (.00–.800)	.857 (.671–.999)
	Random Forest	.733 (.556–.858)	.714	.739	.455	.895	.556 (.200–.778)	.764 (.540–.988)
	SVM	.800 (.627–.905)	.429	.913	.600	.840	.500 (.000–.800)	.745 (.516–.975)
	Decision Tree	.667 (.488–.808)	.714	.652	.385	.882	.500 (.167–.727)	.742 (.512–.972)
	XGBoost	.767 (.591–.882)	.571	.826	.500	.864	.533 (.182–.779)	.727 (.493–.961)
	AdaBoost	.800 (.627–.905)	.429	.913	.600	.840	.500 (.000–.800)	.717 (.481–.953)
	LightGBM	.600 (.423–.754)	.571	.608	.307	.823	.400 (.111–.636)	.661 (.416–.907)
	Gaussian Naive Bayes	.733 (.556–.858)	.714	.739	.455	.895	.556 (.221–.800)	.658 (.413–.904)
	KNN	.733 (.512–.972)	.286	.870	.400	.800	.333 (.167–.727)	.643 (.395–.890)
Clinical risk scores	CHA_2_DS_2_–VASc	.633 (.455–.781)	.999	.552	.389	.999	.560 (.285–.750)	.761 (.536–.761)
	C_2_HEST	.700 (.521–.833)	.571	.739	.400	.850	.471 (.292–.629)	.727 (.493–.961)
	HAVOC	.667 (.488–.808)	.571	.739	.400	.850	.471 (.133–.715)	.714 (.478–.951)
	CHADS_2_	.667 (.488–.808)	.714	.652	.385	.882	.500 (.167–.720)	.689 (.448–.931)
	HATCH	.467 (.302–.639)	.999	.304	.304	.999	.467 (.22–.651)	.634 (.385–.882)

*Note*: Values in parentheses indicate 95% confidence intervals (CI) where applicable.

Abbreviations: AUC, area under the curve; CI, confidence interval; PPV, positive predictive value; NPV, negative predictive value; SVM, support vector machine; KNN, k‐nearest neighbours.

### 3.4 Evaluation of the clinical risk scores

Among clinical risk scores, CHA_2_DS_2_–VASc, C_2_HEST and HAVOC scores had the highest AUCs of .761 (95% CI .536–.761), .727 (95% CI .493–.961) and .714 (95% CI .478–.951), respectively (Figure 2B). C_2_HEST score had the highest accuracy (.700, 95% CI .521–.833) compared to other clinical risk scores. Key metrics for predictive performance of other clinical risk scores are presented in Table 2.

### 3.5 Exploratory comparisons of machine learning models with clinical risk scores

**FIGURE 4:**
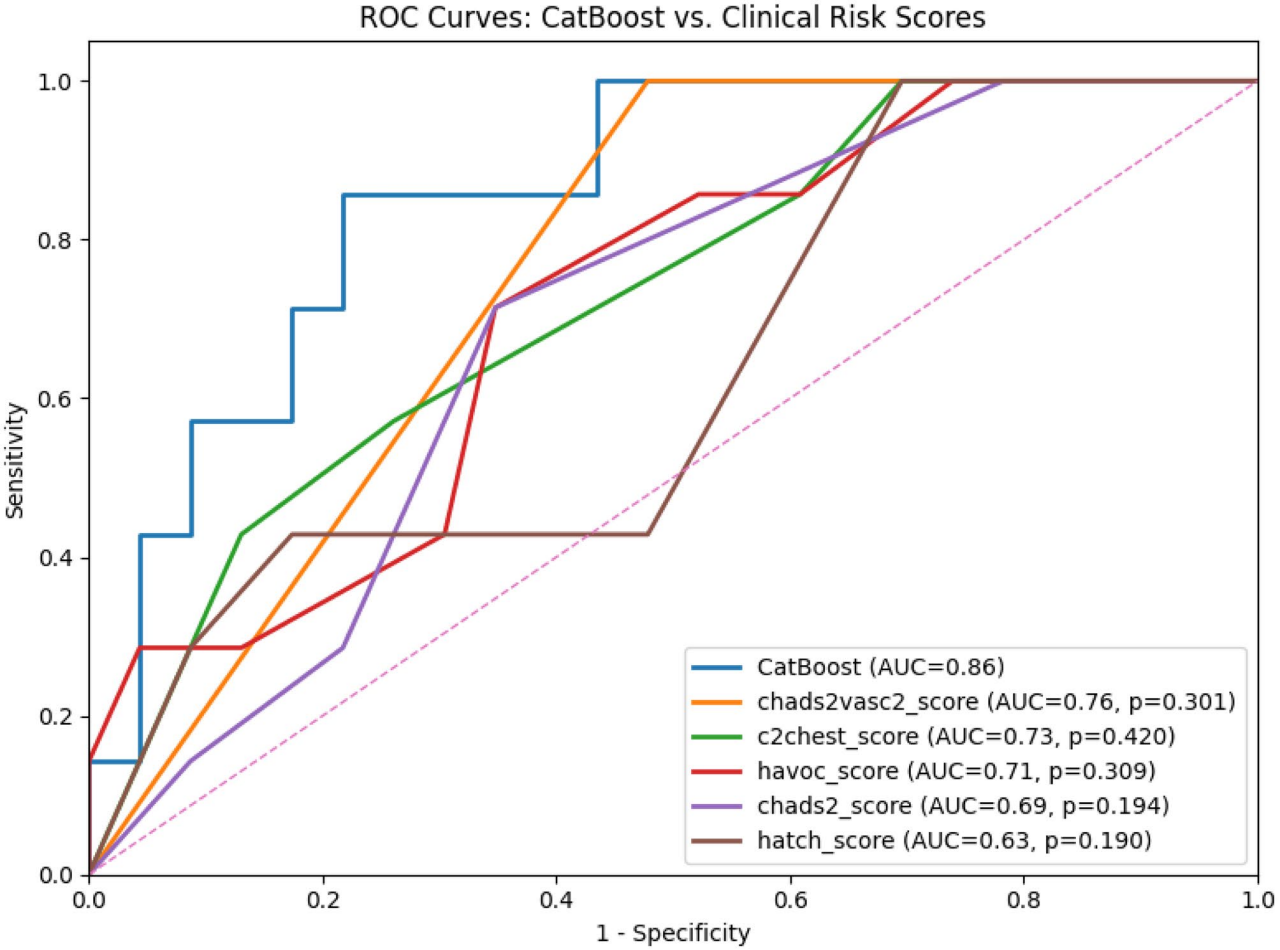
ROC curves for CatBoost model versus clinical risk scores. The *p*‐values demonstrate the significance of the difference of AUCs for each clinical risk score and CatBoost model.

The CatBoost model achieved numerically higher AUC when comparing with clinical risk scores, but differences between the CatBoost model and clinical risk scores were not statistically significant (all *p*‐vales >.05) (Figure 4).

## 4 DISCUSSION

Our principal findings are as follows: (i) one in four patients with AHRE developed AF; (ii) patients with AHRE who developed AF exhibited a clinical phenotype characterised by advanced age and a higher burden of comorbidities, including hypertension, diabetes, coronary artery disease and heart failure; (iii) the CatBoost ML model achieved the highest AUC compared to other ML models and clinical risk scores; and (iv) the most important influential factors in the CatBoost model were LAE, hypertension, diabetes and age.

The incidence of AF observed in this study is consistent with findings from previous studies involving patients with AHREs.^1^, ^20^ In a prospective cohort of 6580 patients with AHRE from the Clinical Significance of Atrial Arrhythmias Detected by Implanted Device Diagnostics [(TRENDS), Italian ClinicalService and Phase IV Long Term Observational Study of Patients Implanted With Medtronic CRDM Implantable Cardiac Devices (PANORAMA)], part of the Stroke Prevention Strategies based on Atrial Fibrillation information from implanted devices (SOS AF) project, 34.1% (*n* = 2244) of the study population developed AF during a follow‐up period of 2.4 ± 1.7 years; notably, they were older and had more diagnosed with heart failure.^20^ In another retrospective observational study from a tertiary care academic hospital in Italy, among 104 patients with AHRE, 29.8% (*n* = 31) progressed to atrial fibrillation, over a median follow‐up of 24.3 months.^1^ Taken together, according to findings from our study and the existing literature, a range of 24%–34% of patients with AHRE develop clinical new‐onset AF, representing a substantial proportion.

Another important finding of our study was that patients who developed AF had a significantly higher burden of comorbidities, including hypertension, diabetes, coronary artery disease and heart failure. There is limited data regarding the comorbidity difference among AHRE patients who developed AF or not. The only study that have been investigated this reported no significant difference among comorbidities among AHRE patients who developed AF compared to those who did not.^20^ However, there is a strong body of evidence demonstrating that comorbidities, including hypertension,^21^ diabetes,^22^ coronary artery disease^23^ and heart failure^24^ are associated with a higher risk of developing new‐onset clinical AF compared to patients without mentioned comorbidities. Further larger studies are warranted to identify comorbidity differences among patients with AHRE who are at risk of AF since it would be helpful in risk stratification and implementing preventive strategies.

Of the clinical risk scores evaluated in our study, CHA_2_DS_2_–VASc score achieved the highest AUC in predicting new‐onset clinical AF (.761, 95% CI .536–.761), which was numerically lower AUC but not statistically significant to the CatBoost ML model, in our exploratory analysis. There are limited data regarding the association of clinical risk scores with developing new‐onset clinical AF in patients with AHRE.^1^, ^8^, ^20^ For example, in a study analysing 470 patients with CIED and without a history of AF, 5 clinical risk scores were assessed for their predictive value: CHA_2_DS_2_‐VASc score, C_2_HEST score, mCHEST score, HAT_2_CH_2_ score and HAVOC score. Of these, only HAT_2_CH score was found to be an independent predictor of new‐onset AF in patients with CIEDs.^8^ Similarly, in a prospective multicentre study analysing 6580 CIED patients without a history of AF and OAC use, 2244 (34.1%) of patients had AHRE over a mean follow‐up of 2.4 ± 1.7 years, and age, male gender and CHADS_2_ score were independent predictors of progression to a daily AHRE burden of more than 23 h, which in the multivariable analysis, age was the only significant predictor.^20^ Imberti et al. demonstrated that CHA_2_DS_2_‐VASc score was identified as independent predictors of progression to a daily AHRE burden exceeding 23 h; however, on multivariable analysis, age remained the only significant predictor.^1^ While clinical risk scores such as HAT_2_CH_2_ and CHA_2_DS_2_‐VASc score have been associated with a higher risk of developing clinical new‐onset AF, their predictive performance remains modest. This is likely due to their limited ability to capture the complex interplay of factors present in this high‐risk, comorbidity‐rich CIED population.

In our study, the CatBoost ML model achieved the highest AUC among all ML models and clinical risk scores. To the best of our knowledge, this is the first study to implement ML models to identify patients with AHRE who are at higher risk of AF. Notably, ML has shown promising results in predicting new‐onset AF in the general population.^9^, ^10^, ^25^, ^26^ In a prospective diagnostic study on 2,252,219 individuals, ML models were applied to predict new‐onset AF within 6 months, achieving an AUC of .800 —slightly outperforming a logistic regression model based on established clinical risk factors, which achieved an AUC of .794.^10^ In another study, deep learning techniques successfully distinguished AF from atrial tachycardia in patients with CIED, with an AUC of .97.^25^ Furthermore, deep learning techniques applied to ECGs demonstrated strong predictive performance for incident AF in a cohort of 145,323 patients, with an AUC of .78 in the internal cohort and .77 in the external cohort.^9^ These findings suggest that ML can play a pivotal role in predicting AF, particularly given that preventive strategies are more effective when targeted at high‐risk individuals than when initiated after the onset of AF.

In our study, LAE was the most important feature in the CatBoost prediction model. Moreover, mild to severe LAE was more frequently observed in patients who progressed to clinical AF. Recent studies have reported associations between atrial cardiomyopathy and both AHRE and clinical AF.^27^, ^28^, ^29^ These findings suggest that precise echocardiographic assessment of the left atrium may aid in risk stratification of patients with AHRE, particularly those at higher risk of progressing to clinical AF.

Additionally, age, hypertension and diabetes—all components of the CHA_2_DS_2_‐VASc score—were among the top four most important features of the CatBoost model for predicting new‐onset AF in AHRE patients in our cohort. This is in agreement with previous studies, which have shown that age, CHADS_2_ and CHA_2_DS_2_‐VASc score are significant predictors of new‐onset AF in patients with AHREs.^1^, ^20^

Patients with CIEDs are usually older patients with multiple clinical comorbidities leading to cardiac arrhythmias.^30^ The associations between hypertension and diabetes and an increased risk of AF are well established in the literature.^31^, ^32^ It is therefore reasonable to consider that comorbidities such as hypertension and diabetes, along with advanced age, play an important role in the development of AF in patients with AHRE and contribute themselves to increasing the risk of cardiovascular death, stroke and systemic embolism in these patients.^33^, ^34^

Taken together, these findings support the hypothesis that patients with AHRE—particularly those at higher risk—may benefit from comorbidity management as part of the holistic or integrated care approach to AF management, which has been associated with significantly improved outcomes.^35^, ^36^ Hence, in high‐risk patients with AHRE, integrated care management should be encouraged—not only to reduce the risk of adverse events but also to lower the likelihood of progression to clinical AF. Indeed, the presence of AHRE may confer an increased risk of stroke necessitating oral anticoagulation, especially where vascular disease, high CHA_2_DS_2_‐VASc score or prior stroke are evident.^37^, ^38^, ^39^

Based on our findings, a personalized management approach—including targeted comorbidity management and detailed echocardiographic evaluation of the left atrium—could be considered for patients with AHRE. In particular, older patients with comorbidities such as hypertension, diabetes and LAE may warrant closer follow‐up and more intensive risk factor management.

## 5 LIMITATIONS

Our study was a single‐centre investigation with a limited sample size and short follow‐up duration, which may restrict the generalizability of our findings. Although the ML models were trained and tested using randomly divided datasets,^1^ it is important to note that a small number of events relative to the number of predictors may have caused some overfitting in our study, which can be mitigated by future external validation in independent cohorts to assess the generalizability and robustness of the model. Although AHRE duration has been reported to be associated with AF progression, we did not include this variable in our models because it was significantly higher in all patients who developed the outcome. Including it would likely have led to significant model overfitting, particularly in the context of our small sample size and limited number of events.

Additionally, the broader applicability of our results to different populations may be limited, especially given the reported ethnic differences in the clinical epidemiology of AF as well as AF‐related complications, such as stroke.^40^, ^41^ Given the limited numbers, our comparison with clinical scores was exploratory, and the limitations of focusing only on the AUC to compare risk prediction scores are well recognised.^42^ Additionally, due to the observational nature of our study, we cannot establish causal relationships between the identified predictors and the development of AF. Future studies with larger, multi‐centre cohorts and prospective validation are needed to further assess the robustness and clinical utility of our models.

## 6 CONCLUSION

ML techniques are robust in predicting new‐onset AF in patients with CIEDs experiencing AHREs. A comprehensive approach—including management of comorbidities, closer follow‐up and focused echocardiographic assessment of the left atrium—may aid in identifying and managing AHRE patients at increased risk of progression to clinical AF.

## AUTHOR CONTRIBUTIONS

Amir Askarinejad: formal analysis, methodology, writing—original draft, writing—review and editing, visualization; Tommaso Bucci, Enrico Tartaglia, Michele Rossi and Yang Chen: writing—review and editing; Niloofar Asgharzadeh and Zahra Amirjam: data curation and investigation; Majid Haghjoo and Yalin Zheng: supervision; Gregory Y. H. Lip: conceptualisation, methodology, project administration and supervision.

## CONFLICT OF INTEREST STATEMENT

GYHL is a NIHR Senior Investigator and coprincipal investigator of the AFFIRMO project on multimorbidity in AF, which has received funding from the European Union's Horizon 2020 research and innovation programme under grant agreement No 899871. All other authors have reported that they have no relationships or conflict of interest relevant to the contents of this paper to disclose.

## Supporting information


Appendix S1.

## Data Availability

The data underlying this study will be made available upon reasonable request to the corresponding author.
