# Supplementary material for: Machine learning‐based prediction of atrial fibrillation in patients with atrial high‐rate episodes
Source: Eur J Clin Invest. 2025 Sep 14;56(1):e70121. doi: 10.1111/eci.70121 (PMC12811835; doi:10.1111/eci.70121)
Supplement: Supplementary file 1 — Appendix S1. [file ECI-56-e70121-s001.docx]

**Machine Learning-Based Prediction of Atrial Fibrillation in Patients with Atrial High-Rate Episodes**

Amir Askarinejad^1^, Tommaso Bucci^1,3^, Niloofar Asgharzadeh^2^, Zahra Amirjam^2^, Enrico Tartaglia^1^, Michele Rossi^1^, Yang Chen^1^, Yalin Zheng^1^, Gregory Y. H. Lip^1,4,5^**^*^**, Majid Haghjoo^6,7^**^*^**

Supplementary material

136 patients were initially evaluated

100 patients were included in the final analysis.

12 patients excluded because history of AF and 7 because of history of AF-related symptoms

17 Patients excluded because of unavailable medical records

Figure S1. Flowchart of the study.

Table S1. Cut-Off Thresholds and Corresponding Youden Index Values for Clinical Risk Scores.

| Clinical risk score | Cut-off | Maximized Youden index |
| --- | --- | --- |
| **CHA_2_DS_2_–VASc** | 3.0 | 0.522 |
| **C_2_HEST** | 3.0 | 0.310 |
| **HAVOC** | 5.0 | 0.366 |
| **CHADS_2_** | 2.0 | 0.366 |
| **HATCH** | 1.0 | 0.304 |
